# Exploring the impacts of producer services agglomeration on manufacturing carbon emissions: Empirical evidence from China

**DOI:** 10.1371/journal.pone.0310527

**Published:** 2024-09-30

**Authors:** Yuping Yang, Lujuan Ye, Jiahe Liu, Xiaoyan Zhang, Johnny F. I. Lam, Huangxin Chen, Ka Leong Chan

**Affiliations:** 1 School of Management, Putian University, Putian, China; 2 School of Economics, Fujian Normal University, Fuzhou, China; 3 Faculty of Humanities and Social Sciences, Macao Polytechnic University, Macao, P R China; Tsinghua University, CHINA

## Abstract

This study employs panel data from 30 provinces and cities in China from 2004 to 2019 to empirically estimate the relationship between producer services agglomeration (PSA) and manufacturing carbon emissions. The findings suggest that such agglomeration is beneficial for lowering carbon emissions in manufacturing, and this conclusion passes multiple robustness tests. Heterogeneity analysis results show that PSA in the east and west regions significantly lowers manufacturing carbon emissions, while its impact in the central region is not significant. High-end PSA is beneficial for cutting carbon emissions in manufacturing, but the inhibitory effect of middle- and low-end PSA is not significant. PSA significantly suppresses carbon emissions from capital- and technology-intensive manufacturing, while it has little impact on carbon emissions from labor-intensive manufacturing. Further analysis reveals that PSA has a dual-threshold impact based on absorptive capacity and a single-threshold effect based on infrastructure level on manufacturing carbon emissions. As the absorption capacity crosses the second threshold or the infrastructure level crosses the first threshold, the inhibition effect of PSA on manufacturing carbon emissions begins to become prominent and shows a trend of enhancement. Our research findings provide theoretical and empirical bases for lowering carbon emissions in the manufacturing sector and fostering its ascent to the highest position of the value chain.

## 1. Introduction

China’s economy in recent years has developed tremendously, but along with it, carbon dioxide emissions have been continuously rising, causing more serious environmental problems [1]. The BP World Energy Statistical Yearbook 2022 states that China’s primary energy consumption accounted for 26.5% of the world’s total primary energy consumption in 2021, and its carbon emissions were 30.7% of global carbon emissions, making it the largest energy consumer and carbon emitter country. As a pillar industry of its economic development, the manufacturing industry has not yet abandoned its growth path of excessive energy use, leading to a sharp rise in carbon emissions.

In fact, carbon reduction in the manufacturing sector is a key priority for the Chinese government in its efforts to combat climate change and promote sustainable development. In 2020, its Implementation Plan for Carbon Peak in the Industrial Sector emphasized constructing a green manufacturing system, quickening the industry’s shift to a low-carbon, green economy, and fostering high-quality growth. In 2021, the Notice on the 2030 Carbon Peaking Action Plan was issued, which clearly stated the main goals of promoting carbon peaking action and focused on implementing ten major actions, including carbon peaking in the industrial sector and the transition to low-carbon energy. How to effectively cut carbon emissions in the manufacturing industry and explore emission reduction paths that comply with the development laws of this industry have emerged as pressing practical issues that require immediate resolution.

As the division of labor continues to deepen and global value chains develop quickly, the producer service sector is completely integrated into the manufacturing production process through input-output relationships, bringing closer the economic connection between the two [2]. The producer services sector exhibits low energy usage, low pollution, and high knowledge intensity as an intermediate input factor [3], which can offer high-end service element support, including expertise, technology, and information, for the manufacturing industry’s production process, and is a key factor in China’s manufacturing sector’s efforts to reach the upper echelons of the value chain. China’s producer service sector has advanced rapidly in recent years, forming a development model characterized by industrial agglomeration.

Studies have indicated that producer services agglomeration (PSA) influences manufacturing productivity through various means, including elevating the level of specialization [4, 5], reducing transaction costs [6, 7], prolonging the value chain in industry [8, 9], and improving technological innovation level [6, 10]. Given the resource environment constraints and the tightening regulations on carbon emission reduction, the question of whether and how such agglomeration can enhance efficiency in energy use and minimize carbon emissions associated with manufacturing. This issue is clearly worth further exploration. The study thus investigates the associations between PSA and manufacturing carbon emissions.

The following features of this study represent its primary innovations and contributions. First, other studies have concentrated more on how PSA affects economic growth, manufacturing productivity, manufacturing technology progress, and overall environmental performance and given less concern to the correlation between such agglomeration and manufacturing carbon emissions. Because of this, we investigate the problem of reducing carbon emissions during the manufacturing development process and conduct a thorough analysis of the PSA’s internal mechanism impacting manufacturing carbon emissions, which is helpful to supplement and deepen existing research content.

Second, this paper looks at the heterogeneous effect of PSA on manufacturing carbon emissions at the industry and regional levels, providing new empirical evidence. At the regional level, we mainly evaluate the connection between PSA and manufacturing carbon emissions under different economic development levels. At the industry level, it addresses the influence of PSA on carbon emissions of various intensive manufacturing industries as well as how the manufacturing industry’s carbon emissions are impacted by the different producer service types.

Third, the external constraints of PSA affecting manufacturing carbon emissions are considered herein. PSA’s impact on manufacturing carbon emissions is a dynamic, complex process that is readily affected by outside variables, including regional absorption capacity and infrastructure construction level. Therefore, absorption capacity and infrastructure construction level are included in the analysis framework to further investigate the non-linear threshold influence of PSA on manufacturing carbon emissions. Doing so helps improve the shortcomings of other literature in describing the relationship between the two.

This paper’s remaining content runs as follows. In the second section, pertinent literature is reviewed and arranged. An in-depth explanation of the variable data and econometric model is given in the third section. The empirical findings of the paper are examined in the fourth section. The fifth section summarizes the research findings, along with recommendations for related policies.

## 2. Literature review

### 2.1. Research on the impact of PSA on economic growth

There are presently two main views on the correlation between PSA and economic development. The first viewpoint holds that such agglomeration fosters economic growth. The majority of academics noted that the concentration of labor and knowledge sharing that result from an industry’s agglomeration in a particular area typically drive productivity growth [11]. A greater agglomeration economy and a notable increase in the productivity of the industrial sector can result from the specialization of producer services, as stated by Rivera-Bratiz [12]. Zhang [13] thought that one significant factor contributing to the rise in urban productivity is the agglomeration of knowledge-intensive service businesses. Song et al. [14] noted that PSA is able to stimulate economic development through knowledge and technology spillover. The second viewpoint suggests that PSA does not significantly boost the productivity of cities and may even suppress economic growth. Lv and Ren [15] emphasized that PSA exerts a threshold impact on raising urban productivity and that PSA is currently not at a level that is stimulating productivity development in the majority of Chinese cities located in the Yangtze River Delta. Zheng and He [16] argued that the polarization effect of PSA causes a concentration of different creative components in core cities, which is detrimental to the economic growth of neighboring cities.

Furthermore, a few academics have also talked about the economic implications of the agglomeration of various forms of producer services. Du et al. [17] suggested that the diversification of PSA is beneficial for enhancing the resilience of economic development, while the influence of specialized agglomeration is contrary. You [18] discovered that PSA can effectively improve urban productivity, but the effects of various agglomeration patterns vary greatly. The promotion role played by specialized agglomeration in the east is more evident, whereas the promotion influence of diversified agglomeration in other regions is more obvious. Peng et al. [19] discovered that high-end PSA is positively related to urban economic performance, while low-end PSA has no significant promoting effect.

### 2.2. Research on the impact of PSA on manufacturing industry development

Most academic studies on the connection between PSA and the development of manufacturing primarily focus on this industry’s productivity and technical level. Eswaran and Kotwal [20] recognized that expanding the service sector would effectively decrease the cost of service factor input into industrial production, hence contributing to the improvement of industrial productivity. According to Simmie and Strambach [21], the division of knowledge and advancement of manufacturing technology levels are fostered by the consolidation of knowledge-intensive service sectors. Zhang [4] confirmed that the specialization of PSA increases manufacturing labor productivity in the eastern and western areas of China but has a restraint in the central area, while the diversification of PSA has the opposite effect. Lanaspa et al. [22] mentioned that producer services and manufacturing co-locate to a large extent, and PSA is an important factor for advancing manufacturing productivity. Yang et al. [23] believed that PSA represents a novel means of increasing productivity in the manufacturing sector. Moreover, talent, technology, and infrastructure sharing from PSA contribute to lower related costs, thereby raising manufacturing efficiency [6]. Gao et al. [24] discovered that PSA is beneficial for improving the technological complexity of export manufacturing. However, Ke et al. [25] asserted that the manufacturing and producer service sectors have an interdependent relationship, with the former typically locating in cities where the latter does.

### 2.3. Studies on the impact of PSA on environmental performance

Apart from its effects on economic growth and manufacturing industry development, the influence studies on environmental performance of PSA are increasing, and two main viewpoints have been formed. The first viewpoint emphasized that the PSA is conducive to improving environmental performance [26, 27]. Du and Zhang [28] stated that PSA promotes green economy development through two paths of green technology advancement and industrial upgrading, and this positive effect will be affected by industrial policies and environmental regulation policies. Further, other scholars have also considered the spatial relevance of PSA. Yang et al. [29] and Yang et al. [30] argued that PSA encourages the enhancement of environmental performance in nearby and local areas by utilizing the spatial spillover effect. The second viewpoint is that PSA has a negative impact on environmental performance. He et al. [31] revealed that PSA does not exert agglomeration effects and is not boosting the green total factor productivity of cities. Moreover, some scholars offered that the specialization of PSA is significantly detrimental to boosting the environmental performance of nearby regions based on the different modes of PSA [32]. In the past few years, governments have blindly pursued high-level industrial agglomeration, resulting in industry incompatibility and inadequate efficiency. As a result, the diversification of PSA impedes not only the benefit of local green development [33] but also the enhancement of environmental quality in the surrounding areas [34, 35].

The association between PSA and carbon emissions has also attracted the attention of scholars. Some scholars believed that PSA has an emission reduction effect [7]. Zhang et al. [36] stated that PSA efficiently promotes the entry of creative new firms, which increases the effect of carbon emission reduction. Zhao et al. [37] discovered that PSA mitigates carbon emissions by maximizing industrial structure and fostering technology and knowledge spillovers. However, according to some research, the association between PSA and carbon emissions is not linear [38]. Ma and Yao [5] revealed that the distribution, structure, and technology effects contribute to the U-shaped connection between PSA and carbon emission efficiency, both locally and in nearby regions. Xu et al. [39] verified that collaborative agglomeration and carbon productivity have a U-shaped connection, which implies that increasing collaborative copolymerization is necessary to boost urban carbon productivity. In addition, some literature has begun to examine the association between producer service subsectors growth and carbon emissions or carbon productivity, especially in the financial services industry [40, 41]. For example, Sun et al. [42] found that digital finance considerably raises the cities’ carbon productivity through marketization and human capital effects, with a spatial spillover effect between the two.

## 3. Econometric model and variable description

### 3.1. Model setting

This research aims to determine whether PSA can lower carbon emissions in manufacturing, which requires accurate identification of the causal relationship between the two. As a result, we create a panel data model in accordance with the analysis above to investigate how PSA affects manufacturing carbon emissions. The regression model is:

$$\ln\;yzco2_{it}=\alpha_{0}+\alpha_{1}agg_{it}+\Gamma X_{it}+\delta_{i}+\gamma_{t}+\varepsilon_{it},$$ (1)

where province and year are represented, respectively, by *i* and *t*; ln *yzco*2 is the manufacturing-related carbon emission level; *agg* denotes the level of PSA; *X* is control variables; *α*_0_ represents the constant term; *α*_1_ denotes the main estimated coefficient; *Γ* denotes the matrix of coefficients for the control variables; *δ*_*i*_ and *γ*_*i*_ denote the regional effect and time effect, respectively; and the random perturbation term is shown by *ε*_*it*_.

External factors may impose limitations on the consequences of PSA on manufacturing carbon emissions, leading to a non-linear correlation between the two variables. Therefore, this study draws on Hansen [43] and selects absorptive capacity and infrastructure as threshold variables, respectively, so as to construct the threshold regression model for the influence of PSA on manufacturing carbon emissions. This specific form is:

$$\begin{array}{c} \ln yzco2_{it}=\beta_{0}+\beta_{1}agg_{it}\cdot I(Q_{it}\leq \xi_{1})+\beta_{2}agg_{it}\cdot I(\xi_{1}<Q_{it}\leq \xi_{2})\\ +\beta_{3}agg_{it}\cdot I(Q_{it}>\xi_{2})+\Pi Z_{it}+\chi_{i}+\tau_{it} \end{array},$$ (2)

where *Q* denotes the threshold variable, including absorptive capacity and infrastructure variables; *ξ* stands for the estimated threshold; *I*(⋅) is the indicator function; *Z* denotes control variables; *β*_0_ denotes the constant term; *β*_1_, *β*_2_, and *β*_3_ are estimated coefficients; *Π* denotes the matrix of coefficients for the control variables; and the random perturbation term is represented by *τ*_*it*_.

### 3.2. Variable descriptions and data sources

#### 3.2.1 Manufacturing carbon emissions

Considering the data availability, we mainly select 27 manufacturing industry segments as the research object and use the logarithmic value of the sum of carbon emissions of different industries as the indicator to measure the carbon emissions of the manufacturing industry.

#### 3.2.2. Producer services agglomeration

Since location quotient can more accurately reflect the spatial distribution of industries and components, most studies adopt it to examine the degree of industrial agglomeration or economic agglomeration. For this reason, we primarily adopt location quotient to assess the level of PSA. The methods are as follows:

$$agg_{ij}=\left(\frac{e_{ij}}{\sum_{j}e_{ij}}\right)/\left(\frac{\sum_{i}e_{ij}}{\sum_{i}\sum_{j}e_{ij}}\right),$$ (3)

where *e*_*ij*_ denotes employment in producer services sub-sector *j* in region *i*; $\sum_{j}e_{ij}$ denotes employment in all industries in region *i*; $\sum_{i}e_{ij}$ denotes employment in producer services sub-sector j in all regions; and $\sum_{i}\sum_{j}e_{ij}$ denotes employment in all industries in all regions.

#### 3.2.3. Control variables

Regression modeling also takes into account the primary control variables influencing manufacturing-related carbon emissions, including economic development level, education level, infrastructure level, industrial structure upgrading, opening to the outside world, and fiscal decentralization. The degree of regional economic growth (*lnpgdp*) is calculated using the logarithm of GDP per capita. Educational attainment (*edu*) is mainly measured using years of education per capita. The highway mileage to administrative area ratio is the one used to evaluate each region’s infrastructure level (*infrac*). The measurement index for industrial structure upgrading (*industry*) is the proportion of the tertiary industry’s value added to that of the secondary industry. The ratio of real foreign direct investment utilization to regional GDP, adjusted for the RMB exchange rate for that year in the calculation procedure, is used to quantify the degree of openness to the outside world (*fdi*). The local general public budget’s expenditure to revenue ratio serves as a gauge for fiscal decentralization (*gova*).

#### 3.2.4. Data sources

We selected panel data from 30 Chinese provinces from 2004 to 2019 (excluding Tibet, Taiwan, Hong Kong, and Macao) for the test due to data availability, and a few values that are absent are filled in using interpolation. Data on manufacturing carbon emissions is acquired from the China Carbon Accounting Databases (CEADs). The China Statistical Yearbook, China Environmental Statistical Yearbook, and statistical yearbooks of different provinces are the sources of data for the remaining variables. In addition, we set the base period to 2003 and adjusted all relevant variables to the constant price in order to guarantee data comparability between years. The primary variables’ descriptive statistics are displayed in Table 1.

**Table 1:** Descriptive statistical analysis.

Variable	N	Mean	Std Dev	Min	Max
*lnyzco2*	480	4.1557	0.9141	1.2146	6.1708
*agg*	480	1.0068	0.3331	0.6354	2.5613
*lnpgdp*	480	10.0492	0.6222	8.3090	11.6299
*edu*	480	8.8017	1.0068	6.3778	12.7820
*infrac*	480	1.1311	1.6453	0.0389	11.6657
*industry*	480	0.7445	0.3035	0.3919	2.1342
*fdi*	480	0.0226	0.0178	0.0001	0.0819
*gova*	480	2.2810	0.9666	1.0517	6.7450

## 4. Empirical results and analysis

### 4.1. Baseline regression results

Table 2 presents the regression results on PSA’s effect on manufacturing carbon emissions. The Hausman test results show that fixed effects are preferable to random effects, rejecting the original assumption of random effects at the 1% statistical level. Therefore, a regression test is performed in this study utilizing a two-way fixed effect model.

**Table 2:** Baseline regression results.

Variable	(1)	(2)	(3)
	Fixed effect	Fixed effect	Random effect
*agg*	-1.1479**	-0.5819**	-0.5844***
	(0.5166)	(0.2356)	(0.2238)
*lnpgdp*		0.9101	0.8406
		(0.6263)	(0.5392)
*edu*		-0.3108*	-0.2899*
		(0.1739)	(0.1698)
*infrac*		0.1529***	0.1329***
		(0.0448)	(0.0339)
*industry*		-0.9560***	-1.0276***
		(0.3155)	(0.2385)
*fdi*		-4.6629*	-4.5479*
		(2.5504)	(2.5373)
*gova*		-0.0799	-0.1294***
		(0.0471)	(0.0369)
*_cons*	4.7080***	-0.7979	-0.1408
	(0.5518)	(4.6275)	(3.6365)
Time fixed effect	Yes	Yes	Yes
Regional fixed effect	Yes	Yes	Yes
Hausman test		21.64 [0.0056]	
Observations	480	480	480
R-squared	0.5990	0.7325	0.7312

Notes: *, **, and *** indicate significance at the 10%, 5%, and 1% statistical levels, respectively. () is the T statistic. [] is the F statistic. The follow-up table is the same.

From columns (1) and (2) in Table 2, the regression consequence of PSA is notably negative at the 5% statistical level, meaning that PSA has a suppressive influence on manufacturing carbon emissions. To bolster the credibility of the findings, this manuscript additionally provides the outcome of random effects estimation, displayed in Table 2‘s column (3). The estimation’s findings also demonstrate the existence of a significant negative correlation. This result is a further deepening at the industry level based on the outcomes of Zhao et al. [37], Du and Zhang [28], and Luo et al. [44]. This is because, as an intermediate input, PSA has an influence on manufacturing carbon emissions through a scale effect, knowledge spillover effect, and structural effect [6]. First, from the perspective of scale effect, PSA reduces manufacturing production costs, transaction costs, and pollution control costs through labor and intermediate goods sharing [45, 46], thus committed to realizing a low-carbon production model [38]. Second, such agglomeration accelerates the spillover and diffusion of advanced technologies, promotes the integration of cleaner production technologies and green innovation concepts into the manufacturing production process, and helps to enhance the sector’s green technology level and energy utilization efficiency [6, 38, 47, 48]. Third, PSA can reduce the manufacturing’s demand for polluting energy and lead it to move up the value chain, thereby lowering its level of carbon emissions [36, 49, 50].

### 4.2. Endogenous and robustness tests

#### 4.2.1. Endogenous test

Given the correlational characteristics between industries, PSA possesses an impact on the manufacturing sector’s carbon emissions. Conversely, the rise in its carbon emissions forces the optimization of the industrial production process, generates more demand for high-end service elements, and further promotes the expansion of the scope of PSA. Therefore, this article adopts the first-order lagged term of PSA as the instrumental variable for 2SLS regression, building on the findings of Liu et al. [6] to tackle potential endogeneity issues. The outcomes appear in Table 3.

**Table 3:** Endogenous and robustness tests.

Variable				
	Instrumental variable regression	Replace the explained variable	Add new control variable	Shrink tail test
*agg*	-0.8264***	-0.6474**	-0.6402***	-0.6495**
	(0.2356)	(0.2501)	(0.2206)	(0.2562)
*lnpgdp*	0.9244***	0.4385	0.9538	0.9889
	(0.2636)	(0.6485)	(0.6842)	(0.7860)
*edu*	-0.2859***	-0.3181*	-0.3210*	-0.7210*
	(0.0756)	(0.1847)	(0.1721)	(0.4122)
*infrac*	0.1425***	0.1403***	0.1603***	-0.0741
	(0.0237)	(0.0477)	(0.0430)	(0.0527)
*industry*	-0.8180***	-0.0540	-1.0480***	-4.4940
	(0.2420)	(0.2990)	(0.3316)	(2.7379)
*fdi*	-4.7485***	-4.4416*	-4.7055*	0.1649***
	(1.2182)	(2.6080)	(2.5972)	(0.0527)
*gova*	-0.1191***	0.0043	-0.1051*	-0.2163
	(0.0446)	(0.0388)	(0.0604)	(0.1444)
*lncontrole*			-0.1880	
			(0.3682)	
*magg*			-0.1506	
			(0.1090)	
*_cons*	-0.6585	-4.4512	-0.4928	-2.4577
	(2.4720)	(4.7372)	(4.9325)	(6.3473)
Kleibergen-Paap rk LM statistic	94.357			
	[0.0000]			
Cragg-Donald Wald F statistic	499.544			
	<16.38>			
Time fixed effect	Yes	Yes	Yes	Yes
Regional fixed effect	Yes	Yes	Yes	Yes
Observations	450	480	480	480
R-squared	0.9641	0.7424	0.7339	0.7153

Notes: [] is the p-values; < > is the 10% maximum IV size.

In the first step in regression, the association between instrumental factors and PSA is considerably positive, which supports the correlation hypothesis. The instrumental variables chosen for this paper pass the unrecognizable test, as evidenced by the *p*-value of the Kleibergen-Paap rk LM statistic being less than 0.01 and the null hypothesis being rejected at the statistical level of 1%. The Cragg-Donald Wald F statistic has a value of 499.544, larger than the threshold corresponding to 10%, which means that it passes the weak instrumental variable test. Moreover, the equal number of instrumental variables and endogenous variables indicates that there is no problem of overidentification. In accordance with the aforementioned analysis, the instrumental variables chosen for this paper make sense. After overcoming the endogeneity problem, Table 3‘s column (1) shows that, at the 1% statistical level, the coefficient of PSA and manufacturing carbon emissions is notably negative. Their relationship is still characterized by negativity.

#### 4.2.2. Robustness test

Table 3‘s Columns (2)—(4) present the robustness test findings conducted on the three approaches. First, we utilize the proportion of manufacturing carbon emissions to industrial added value to indirectly represent the extent of manufacturing carbon emissions and take the logarithm for model estimation. The findings point out that, at the 5% statistical level, the consequence of PSA on manufacturing carbon emissions is notably negative—that is, the PSA assists in suppressing manufacturing carbon emissions. This indicates that the negative connection between the two holds even when the explained variable is substituted. Second, this paper adds environmental regulation and manufacturing agglomeration indicators to control the factors that may affect the outcomes of the regression. Among them, the measure of environmental regulation indicators (*lncontrole*) takes the logarithm of industrial pollution control investment and uses the GDP deflator to de-price it. And the degree of manufacturing agglomeration (*magg*) is evaluated by location quotient. The findings demonstrate that the PSA serves to lower the manufacturing sector’s carbon emissions, which agrees with the baseline analysis findings. Third, we further employ the shrinking tail test to investigate the dependability of the baseline regression findings. It can be found that PSA’s regression value is also significantly negative, which provides more evidence of PSA’s detrimental effects on manufacturing carbon emissions. In conclusion, no matter what method is adopted, the PSA’s coefficient on manufacturing carbon emissions is significantly negative—that is, the conclusion that PSA suppresses manufacturing carbon emissions is reliable.

### 4.3. Heterogeneity test

#### 4.3.1. Regional heterogeneity

Owing to the imbalanced advancement of the region, we categorize the sample into the eastern, central, and western regions and proceed with a sub-regional analysis on how PSA affects manufacturing carbon emissions. The outcomes appear in Table 4. Notably, at the 5% statistical significance level, the PSA’s coefficients on manufacturing carbon emissions in both the eastern and western regions exhibit significant negativity. This suggests that PSA acts as a suppressant for manufacturing carbon emissions in these regions. Conversely, in the central region, there is no discernible link between two. This finding is in contrast to the research of Zhao et al. [37], which holds the view that producer services in the central region have obvious agglomeration advantages and can restrain carbon emissions by lowering the share of the secondary industry, while this study’s findings point out that the low degree of PSA in the central region is an important reason for its insignificant impact on the manufacturing sector’s carbon emission reduction. An explanation arises from the observation that the central region relies more heavily on traditional industries with inadequate consideration for the growth of producer services, causing the PSA to be comparatively low. Simultaneously, the central region exhibits a comparatively restricted integration between the producer services and manufacturing sectors, posing challenges for PSA to effectively contribute to enhancing the production process and energy utilization level of the manufacturing industry.

**Table 4:** Regional heterogeneity analysis.

Variable	(1)	(2)	(3)
	East Region	Central region	West Region
*agg*	-0.5549**	-0.4666	-0.8557**
	(0.2277)	(0.6005)	(0.3585)
*lnpgdp*	1.2302***	0.3100	0.3018***
	(0.3348)	(0.1687)	(0.0786)
*edu*	-0.6205*	-0.0465	0.0331
	(0.3306)	(0.0725)	(0.0914)
*infrac*	0.1903	0.1781	0.1959***
	(0.1638)	(0.1607)	(0.0266)
*industry*	-1.2459***	-1.4510**	-1.8835***
	(0.3371)	(0.4995)	(0.3499)
*fdi*	-3.2290	-3.9231	-1.0268
	(3.0738)	(2.6271)	(6.5606)
*gova*	-0.4509*	-0.0439	-0.0670
	(0.2135)	(0.1616)	(0.0532)
*_cons*	-0.4805	3.1677*	2.6457**
	(1.6887)	(1.4899)	(1.0216)
Observations	176	128	176
R-squared	0.6846	0.7153	0.7958

#### 4.3.2. Producer services industry heterogeneity

Given the extensive presence of firms within China’s producer services sector, variations in knowledge and technology density emerge across distinct sub-sectors, which makes it possible for producer services to have different effects on regional development benefits throughout the agglomeration process. In light of this, inspired by the investigations of Guo et al. [51], this study categorizes information transmission, the computer service and software industry, the financial industry, as well as scientific research and technical services industry as high-end producer service industry. Wholesale and retail, transportation, storage, postal services, leasing, and business services fall under the definition of middle- and low-end producer service industry. We run regression analyses on this foundation to determine how high-end and low-end PSA affect manufacturing carbon emissions. Table 5 contains specifics about the comprehensive results.

**Table 5:** Industry heterogeneity analysis of producer services.

Variable		
	High-end PSA	Middle- and low-end PSA
*agg*	-0.6374**	-0.0930
	(0.2718)	(0.1595)
*lnpgdp*	0.6829	1.0018
	(0.4581)	(0.6733)
*edu*	-0.2720*	-0.3251*
	(0.1364)	(0.1825)
*infrac*	0.1338***	0.1566***
	(0.0408)	(0.0451)
*industry*	-0.9437***	-1.1033***
	(0.2336)	(0.3110)
*fdi*	-3.9548*	-4.2027
	(2.1310)	(2.4807)
*gova*	-0.0848*	-0.0556
	(0.0474)	(0.0573)
*_cons*	1.0594	-1.9740
	(3.5537)	(5.0953)
Time fixed effect	Yes	Yes
Regional fixed effect	Yes	Yes
Observations	480	480
R-squared	0.7505	0.7197

The table demonstrates that high-end PSA exerts a negative effect on the manufacturing sector’s carbon emissions, while the impact coefficient of middle- and low-end PSA is not significant. The findings indicate that manufacturing-related carbon emissions are considerably reduced by high-end PSA but not significantly reduced by middle- and low-end PSA. This outcome is comparable to the research conducted by Li et al. [33], which also obtained similar industry heterogeneity in their study on the correlation between PSA and urban carbon emissions. This is because, unlike the traditional low-end service sector, the high-end service sector’s growth emphasizes the support of technological innovation, model innovation, and process innovation in the industrial chain and industrial system, which can provide a technical foundation for the manufacturing industry to realize low-carbon transformation [49, 52]. Moreover, green innovation industries linked to the improvement of the environment are also subordinate to the high-end services sector, which makes high-end PSA has a greater ability to block manufacturing carbon emissions [33]. Put another way, the development of high-end PSA can decrease the manufacturing sector’s energy consumption while increasing the usage of green high-end intermediate service elements.

#### 4.3.3. Manufacturing industry heterogeneity

Considering the large number of industry categories within the manufacturing sector, it is separated into three categories in this study following research by Han and Yang [53]: capital-intensive, labor-intensive, and technology-intensive. We investigate how various manufacturing industry types’ carbon emissions are affected by PSA. Table 6 presents the findings.

**Table 6:** Analysis of manufacturing industry heterogeneity.

Variable	(1)	(2)	(3)
	Labor-intensive	Capital-intensive	Technology-intensive
*agg*	-0.2630	-0.7950***	-0.7507*
	(0.2661)	(0.2883)	(0.4402)
*lnpgdp*	1.8360***	0.2636	-0.2170
	(0.4778)	(0.9573)	(0.6572)
*edu*	-0.1702	-0.3985	-0.1736
	(0.1240)	(0.2740)	(0.1703)
*infrac*	0.0988	0.1836***	-0.0305
	(0.0604)	(0.0468)	(0.0464)
*industry*	-0.7987**	-1.3649**	-0.0998
	(0.3381)	(0.5273)	(0.4740)
*fdi*	-3.3343	-4.3022	2.7980
	(3.3580)	(3.2434)	(2.9582)
*gova*	-0.1057	0.0319	-0.2473
	(0.1059)	(0.0573)	(0.2256)
*_cons*	-12.0638***	5.6701	4.3789
	(4.2191)	(7.0580)	(6.2410)
Time-fixed effect	Yes	Yes	Yes
Regional fixed effect	Yes	Yes	Yes
Observations	480	480	480
R-squared	0.7081	0.5103	0.0944

According to the table, the coefficients of PSA on carbon emissions of capital- and technology-intensive manufacturing sectors are significantly negative. This implies a noteworthy reduction in the carbon emission levels of these industries resulting from PSA. However, the inhibitory impact on carbon emissions of labor-intensive manufacturing industries is not significant. This might be due to the fact that labor-intensive manufacturing industries in China currently have a low degree of integration with producer services, as well as the fact that PSA is more crucial to expanding the production scale of labor-intensive manufacturing industries, rather than improving their development efficiency through production process improvement and technological progress. Therefore, the contribution of PSA to labor-intensive manufacturing industries’ reduction of carbon emissions is not obvious.

### 4.4. Threshold effect test

We also investigate the non-linear aspects of PSA’s effect on manufacturing carbon emissions using the panel threshold model. Specifically, we choose absorptive capacity and infrastructure construction as threshold variables to find out how different external restrictions affect PSA’s impact on manufacturing carbon emissions. Since human capital level is an essential aspect in determining the absorption of knowledge and technology, we measure absorptive capacity by applying the average number of years of education per capita. This paper examines the significance, number, and specific values of the threshold effects using the Bootstrap resampling method. Tables 7 and 8 present the findings.

**Table 7:** Threshold effect test results.

Variable	Threshold type	*F*-value	*P*-value	Threshold value	95% confidence interval
*edu*	Single	152.89	0.0000	7.3749	[7.2911, 7.4336]
	Double	24.35	0.0867	11.4768	[11.1940, 11.5550]
*infrac*	Single	94.21	0.0000	1.2671	[1.2647, 1.2706]
	Double	18.07	0.4300	1.9779	[1.9085, 1.9924]

**Table 8:** Threshold regression result.

Variable		(1)	(2)
		Threshold variable: *edu*	Threshold variable: *infrac*
*agg*	*edu* ≤ 7.3749	-0.2593	
		(0.172)	
	7.3749 < *edu* ≤ 11.4768	-0.0739	
		(0.153)	
	*edu* > 11.4768	-0.5368***	
		(0.150)	
*agg*	*infrac* ≤ 1.2671		-0.1731
			(0.181)
	*infrac* > 1.2671		-0.4654**
			(0.170)
*lnpgdp*		0.4589***	0.5252***
		(0.089)	(0.108)
*edu*		-0.0796	-0.0885
		(0.088)	(0.095)
*infrac*		0.1857***	0.2048***
		(0.020)	(0.023)
*industry*		-1.2903***	-1.4206***
		(0.255)	(0.323)
*fdi*		-3.3475*	-4.1782**
		(1.848)	(2.005)
*gova*		-0.1115**	-0.1165**
		(0.050)	(0.056)
*_cons*		1.4346**	1.0967
		(0.658)	(0.801)
Observations		480	480
R-squared		0.779	0.750

#### 4.4.1. Absorptive capacity threshold

Table 7 indicates that the F-statistics of the single and double thresholds are significant when absorptive capacity is employed as the threshold variable. Based on absorptive capacity, this suggests that PSA exhibits a double-threshold influence on manufacturing carbon emissions, with threshold values of 7.3749 and 11.4768, respectively. We additionally test the regression of the dual absorptive capacity threshold. Table 8‘s column (1) displays the outcomes of the absorptive capacity threshold estimation.

The findings indicate that PSA’s coefficient on manufacturing carbon emissions is negative but not significant when absorptive capacity is below the threshold figure of 7.3749. PSA’s effect on manufacturing carbon emissions remains negative and not statistically significant once absorptive capacity crosses the first threshold, but its coefficient decreases slightly. This may be because when the absorptive capacity does not reach a particular point, producer service factors are not only difficult to be absorbed by the manufacturing industry, but may also increase the use of traditional energy factors by the manufacturing industry. This makes the expansion scale of PSA unfavorable for manufacturing carbon emissions. As pointed out in the study of Dong and Xia [54], when the absorption capacity is lower than a certain threshold value, even if a large number of knowledge- and technology-intensive businesses are introduced, advanced technologies cannot be truly transformed and put into production activities, which will instead have a detrimental influence on the local green economy development.

The PSA’s coefficient on manufacturing carbon emissions turns notably negative at the 1% statistical level when absorptive capacity reaches the second threshold. Moreover, its absolute value increases, indicating that with further improvement of absorptive capacity, the inhibitory impact produced by PSA on manufacturing carbon emissions begins to emerge and shows a trend of continuous strengthening. It means that the absorption capacity has a lag effect, which has also been recognized by Moralles and Moreno [55] and Duong [56]. A possible explanation is that as the region’s absorption capacity rises, it becomes increasingly capable of applying and internalizing high-end service factors and effectively integrating external technical resources [57, 58], so as to encourage the integration and bonding of manufacturing production with high-tech and high-value producer services, facilitate the embedding of clean and environmentally friendly knowledge and technology into the production process, and assist in achieving the manufacturing sector’s transition to a low-carbon economy [59, 60]. Moreover, as stated by Albert-Morant et al. [61], Aboelmaged and Hashem [62], and Song et al. [63], absorption capacity is also a vital component in influencing the spillover of advanced technologies and the potential of green innovation.

#### 4.4.2. Infrastructure threshold

According to Table 7, when infrastructure serves as the threshold variable, its single-threshold F-statistic is significant, however, the double-threshold F-statistic fails to be significant, indicating that the consequence of PSA on manufacturing carbon emissions has a single-threshold effect based on infrastructure with a threshold value of 1.2671. We further test the single threshold of infrastructure through regression. The discoveries of the infrastructure threshold estimation are presented in Table 8‘s Column (2).

The findings present that the PSA’s coefficient on manufacturing carbon emissions is negative but not significant where the infrastructure level is below the threshold figure of 1.2671. As the infrastructure level crosses the first threshold, the coefficient of PSA on manufacturing carbon emissions becomes notably negative, and the coefficient value grows noticeably before and after the threshold. It indicates that the detrimental influence of PSA on manufacturing carbon emissions becomes gradually significant and has a strengthening trend. This is in agreement with the analysis of Wang et al. [64], which hold that only when infrastructure is above a specific threshold will it positively affect industrial energy efficiency, and it will also have an indirect impact via industrial agglomeration. And the improvement of energy efficiency inevitably reduces its carbon emission level. It is evident that infrastructure construction is a critical threshold that determines how PSA affects manufacturing carbon emissions. On the one hand, infrastructure development shortens the spatio-temporal distance between regions [65, 66], enhances the spillover effect of PSA, expands the supply radius of green service resources [67–69], and brings opportunities for low-carbon transformation of manufacturing industries in distant regions. On the other hand, improving the infrastructure level lowers the expense of transportation and high-end service factor transactions [47, 70], encourages manufacturing companies to employ service factors more frequently, and helps the manufacturing sector advance up the high-end value chain.

## 5. Conclusions and policy recommendations

### 5.1. Conclusions

We build a panel model and a threshold model to examine empirically the impact of PSA on manufacturing carbon emissions. The following are the study’s primary conclusions. First, PSA and manufacturing carbon emissions are negatively correlated, in other words, the former helps to lower the latter’s level of carbon emissions. Using the instrumental variable, replacing explanatory variables, adding new control variables, and applying the truncation approach, this result passes the reliability test.

Second, PSA helps lower manufacturing carbon emissions in the east and west but has no effect in the central region when viewed through the lens of regional heterogeneity. The high-end PSA has a considerable part in reducing manufacturing carbon emissions from the standpoint of industrial heterogeneity within the producer service industry, but the low-end PSA has little effect. PSA considerably reduces the carbon emissions of capital-intensive and technology-intensive manufacturing sectors, but has no discernible effect on labor-intensive manufacturing sectors from the standpoint of industry heterogeneity in the manufacturing sector.

Third, PSA’s influence on carbon emissions from the manufacturing sector shows a double-threshold effect based on absorptive capacity. When absorptive capacity crosses the second threshold, the detrimental influence of PSA on manufacturing carbon emissions begins to emerge and shows a continuously increasing trend. Furthermore, PSA has a single-threshold influence on manufacturing carbon emissions based on infrastructure level. As the infrastructure level rises above the first threshold, PSA’s ability to hinder manufacturing carbon emissions is considerably increased.

### 5.2. Policy recommendations

The research findings presented above serve as the foundation for the policy suggestions that this study makes.

First, our study suggests that PSA is an important factor in restraining manufacturing carbon emissions. Therefore, the state ought to enhance PSA and optimize the spatial layout of agglomeration areas. Specifically, on the one hand, it is essential to strengthen the planning guidance and policy support for the producer service industry and encourage and support related firms to establish cooperative alliances with research institutions and government agencies to accomplish this industry’s cluster development, so as to effectively reduce carbon emissions in manufacturing industries. On the other hand, local authorities must develop producer service clusters according to the resource endowment and industrial advantages of various locations. The above study pointed out that PSA’s inhibitory consequence on manufacturing carbon emissions is more pronounced in the eastern and western regions. Therefore, the eastern region ought to continue to build modern service industry brand parks and actively encourage the agglomeration of high-tech service leading enterprises. The western region needs to focus on developing several characteristic PSA areas in order to stimulate the manufacturing sector’s low-carbon growth. Considering that the carbon reduction generated by PSA in the central region has not yet been highlighted, it is imperative to continue to cultivate PSA areas, prioritize the development of producer service firms that complement the leading industries, and establish a development system of producer services that meets the needs of local industry’s low-carbon transformation.

Second, considering the industry heterogeneity of PSA’s consequences for manufacturing carbon emissions, the differences in the growth of producer services and manufacturing subsectors should be the focus of local governments. On the one hand, since the high-end PSA has a clear inhibitory impact on manufacturing carbon emissions, it is important for local governments to vigorously foster the high-end PSA and increase the use of green high-end service elements in the manufacturing sector. Meanwhile, the government can fully utilize modern information technology to change the way the medium- and low-end producer service industry develops, enhance its specialization level and innovation ability, and then improve its service capacity for the low-carbon manufacturing industry’s growth. On the other hand, due to the varying effects of PSA on carbon reduction in different manufacturing sectors, it is imperative to further reinforce the efficient integration of producer services with capital- and technology-intensive manufacturing sectors and promote low-carbon collaborative innovation in product, technology, and management between producer services and the two categories of manufacturing enterprises. Furthermore, the government ought to lay out PSA areas near labor-intensive manufacturing industries, promote the green collaborative agglomeration of the two, and effectively empower the low-carbon transformation of labor-intensive manufacturing industries.

Third, the above study highlights the importance of absorption capacity and infrastructure in PSA’s efforts to curb carbon emissions from manufacturing, for which local authorities must raise the regional absorptive capacity and infrastructure construction level. Specifically, on the one hand, local authorities need to strengthen the training of high-end and composite talents in producer services, establish a talent training system covering green professional technology, research and development design, green financial services, low-carbon operation and management, and enhance the absorption and digestion ability of green knowledge, so as to strongly empower the low-carbon advancement of the manufacturing sector. On the other hand, local authorities must constantly enhance the transportation infrastructure construction around regional and industrial agglomeration areas and promote the free flow and efficient connection of green elements. Simultaneously, it is indispensable to accelerate the layout and construction of information infrastructure, including 5G and data centers, improve the capacity of producer services in low-carbon information transmission and data processing, expand the spatial radius of green knowledge and technology spillover, and further strengthen PSA’s role in lowering manufacturing carbon emissions.
